# Fatigue Crack Propagation Estimation Based on Direct Strain Measurement during a Full-Scale Fatigue Test

**DOI:** 10.3390/s22052019

**Published:** 2022-03-04

**Authors:** Piotr Reymer, Andrzej Leski, Michał Dziendzikowski

**Affiliations:** 1 Airworthiness Division, Air Force Institute of Technology, 01-494 Warsaw, Poland; michal.dziendzikowski@itwl.pl; 2 Faculty of Mechanical Engineering, Military University of Technology, 00-908 Warsaw, Poland; andrzej.leski@ilot.lukasiewicz.gov.pl; 3 Institute of Aviation, Łukasiewicz Research Network, 02-256 Warsaw, Poland

**Keywords:** crack propagation, full-scale fatigue test, trainer aircraft, stress intensity factor

## Abstract

Military aircraft are subjected to variable loads, which are the main cause of initiation and propagation of cracks in the most stressed locations of the airframe. The aim of a Full-Scale Fatigue Test (FSFT) is to represent actual load conditions in such a way that the results obtained are a good representation of the actual loads and may be used as data that give insight into the development of real fatigue damage in critical locations. The FSFT load spectrum is a generalized depiction of the expected service loads and is designed to give an overall good representation of loads exerted on the airframe’s structural elements during operation. Moreover, the discrete method of load application on the structure (exerting loads with hydraulic actuators rather than pressure fields or inertia loads expected in actual operation) may cause some local effects, which may not be present in operation. The proposed usage of direct strain data from the test include such local effects. Moreover, operational loads may vary between individual aircraft, therefore it is crucial to understand the whole process of fatigue crack onset and development in order to determine safe inspection intervals and thereby mitigate risk. This paper presents crack propagation calculations regarding the development of a crack in a critical location of the PZL-130 “Orlik” TC-II aircraft, discovered during FSFT. The discussed crack was found already developed, hence the information about nucleation and initial propagation of the crack was not available. Therefore, there was a need to recreate the whole propagation process by means of numerical estimations using the FSFT results like location of the crack and total life for model validation. Moreover, in order to gather real load data for calculations a dedicated stain gage was installed on the damaged load path to monitor the actual remote strain in the element during the FSFT. This allowed for the definition of load sequence exerted on the damaged element directly during the test rather than estimating it from the general load conditions of the wing. The calculations allowed for the estimation of crack propagation curves from initiation to critical crack length causing fatal damage. The obtained curves allowed to visualize the crack behavior due to applied load and furthermore define initial and recurring inspection intervals for the entire fleet during operation, which allowed to define which cracks could be found before they reach critical size in order to carry out mitigation actions like repair or replacement of the damaged part. The authors present the methodology for load spectrum development based on direct strain measurements and furthermore crack propagation curves estimation, validated with the actual FSFT results, which allowed to propose nondestructive inspection intervals for future operation.

## 1. Introduction

This analysis was initiated by a critical structural damage found during FSFT of the PZL-130 “Orlik” TC-II military trainer aircraft. Since the discovered damage was fully developed, it was only possible to estimate the total life of the damaged structural element. The PZL-130 “Orlik” is a turboprop military trainer aircraft designed according to safe life approach, meaning that the total safe life for the structure was defined not allowing for any damage to occur in primary structure during operation. Due to the found crack from the maintenance point of view it is essential to know not only where, but also when such crack may occur during operation and when it reaches detectable size. This would allow to define whether crack propagation in considered region is maintainable from the damage tolerance point of view (it is possible to find and monitor crack growth with reasonable time intervals before it reaches critical size and causes fatal failure) [1].

Modern aircrafts are designed according to the damage tolerance methodology [1], which means that whereas cracks are expected to occur during operation, the process of initiation and development should be well established in order to ensure safe operation. Throughout the life of an individual aircraft a series of nondestructive inspections (NDI) is carried out to ensure that cracks are found as soon as they reach detectable sizes and can undergo mitigation repairs, or the damaged part is replaced before they reach critical size [2,3,4]. Such approach is not only limited to aircraft but is also used in other branches of transportation [5,6,7]. In the case of older aircraft (like the PZL-130), the safe life approach was typically used, meaning that no cracks should occur during the whole operation time. This approach is based on the linear accumulation of fatigue damage [8] and due to its conservative approach, resulting from the high scatter ratio of such estimation, results in low fatigue life.

For older aircraft designed according to the safe life approach, it is a common practice to carry out a Full-Scale Fatigue Test (FSFT) to verify the possibility of introducing the damage tolerance approach [9,10,11]. The most comprehensive fatigue test is the actual usage, but due to possible critical damage FSFT are commonly used in order to predict any possible cracking [10,11,12,13] before the aircraft is certified to roll out. As with most tests FSFT is a representation of the actual service load conditions [14,15] and simplifies the complexity of the actual load spectrum by neglecting some effects. Moreover, due to discrete load application FSFT may cause some local effects that are absent from actual service. That said, the load spectrum used in FSFT is usually developed from a well-defined flight profile [9,10,11,12], which is basically a good representation of actual operation loads. Nevertheless, it may differ between individual aircraft or from future operational loads of the whole fleet. A great example of the diversity involved is the Aircraft Structural Integrity Program (ASIP) for the F-16 fighter aircraft, which incorporates users from all over the world operating the aircraft in extremely different conditions. Despite the tremendous work done since the F-16 platform entered service back in 1970s, new structural problems are still being found by versatile operators around the world also due to the variations in load spectra among different users.

On the other hand, the load spectrum prepared for a FSFT, although usually based on Operational Load Monitoring program (OLM) [16,17], is focused on general representation of the flight envelope. Local effects on the structure may cause local effects to the load spectrum which will impact the crack propagation process and the total durability of the considered element, especially when discrete load application is considered and local stress concentrations due to fasteners like in the case of PZL-130 occur.

### Problem Definition

Military aircraft, especially trainers, are subjected to variable load spectra which have a major influence on the onset and propagation of fatigue cracks [18]. Therefore, it is of the utmost importance to define all possible crack locations. Cracks usually occur in locations with stress concentrations due to geometric features, high load regions or where material imperfections occur (production or maintenance damage).

In order to calculate crack propagation in the aircraft structure, several key elements must be defined: detailed geometry of the structure, load spectrum expected during operation, material properties of the damaged structure and the crack propagation model (the crack propagation equation with chosen complexity and possible retardation model), among others. The geometrical data is crucial for the purposes of determining stress concentrations and finally the stress intensity factor along the crack tip, which is a crucial parameter in crack propagation estimations [4,19,20]. Load spectrum is considered the main driver of crack propagation, since variable amplitude loads cause changes in stress levels in the crack tip, triggering crack propagation. It is also important to know the exact material properties of the structure. Manufacturer data sheets are a valuable source of general information on the material properties of alloys; however, verification tests provide not only truly accurate figures but additional data on aspects such as fracture toughness *K_IC_* [21].

The detailed geometry of the structural element described in this paper was supplied by the aircraft manufacturer PZL “Warszawa-Okęcie” in the form of a Computed Aided Design (CAD) model, which was used to create the detailed Finite Element Model (FEM) of the cracking flange with the adjacent structure. Moreover, the aircraft manufacturer provided the global FEM model, used to define the general stress condition. These models were essential for determining the global stress field, stress concentrations and stress intensity values along the propagating cracks.

**Figure 1:**
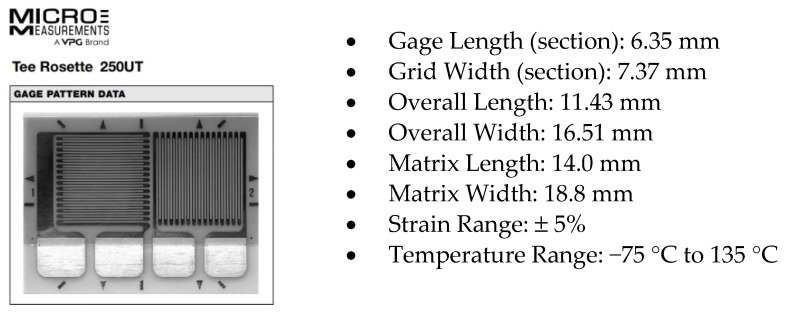
Micromeasurements CEA-250UT 350 Ohm strain gage pattern and technical data.

**Figure 2:**
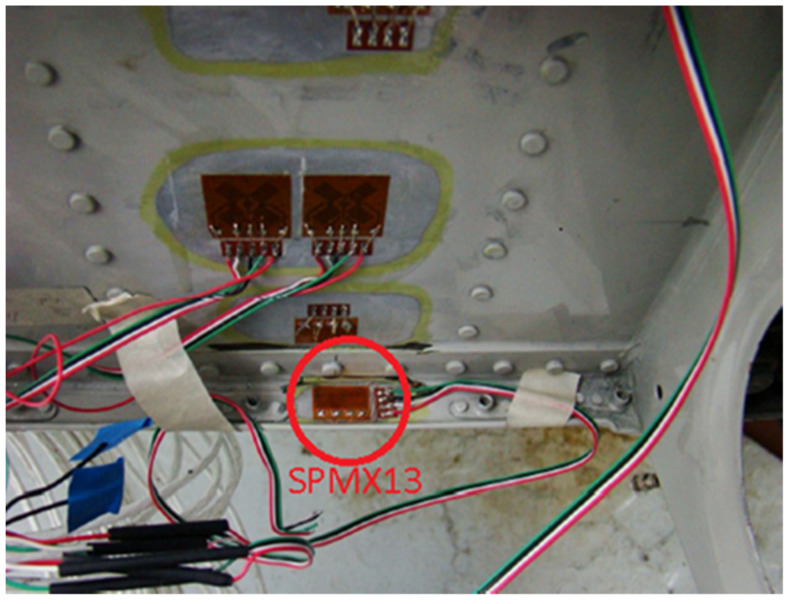
Location of the front spar strain gage SPMX13 used for load spectrum definition.

The load spectrum used in the presented crack propagation calculations was defined using strain measurements recorded during FSFT on the PZL-130 “Orlik” aircraft with a Micro Measurements CEA-250UT 350 Ohm 90° tee rosette strain gage (Figure 1) installed on the front spar lower flange (Figure 2). Data from the front spar was used since the rear spar strain gage was defective, not allowing to gather all the necessary data. However, the properly recorded data was correlated with the front spar gage signal, allowing to define the actual load sequence in the rear spar.

The selected strain gage was one of the strain gages in the array installed throughout the whole test specimen used to validate the models. This ensured that the loads used in the calculations were compliant with the actual loads driving the crack propagation during the test. The FSFT load spectrum was prepared based on the Operational Load Monitoring (OLM) program and historical operational data [17].

Since crack propagation calculation is an iterative method the material properties that feed the crack propagation equation have to be precise, since any discrepancies will accumulate, leading to unrealistic results. Therefore, to assure accurate data additional laboratory tests were carried out on samples manufactured by the aircraft manufacturer using the same aluminum alloys (Al 2024-T4) and manufacturing techniques as during production of the actual aircraft. The key tests were static tension (for the Young’s modulus) and fracture toughness (*K_IC_*) [21].

The crack propagation model used in this work was the NASGRO equation [22], which is a widely used numerical method, capable of determining crack propagation in all three distinguishable crack propagation regimes: initiation, stable propagation and rapid propagation [23]. The crack propagation equation determines the increase of a crack due to the applied load cycle—*da/dN*, where *a* is the crack length and *N* is the number of cycles for the stress range.

## 2. Materials and Methods

### 2.1. Critical Point Definition

**Figure 3:**
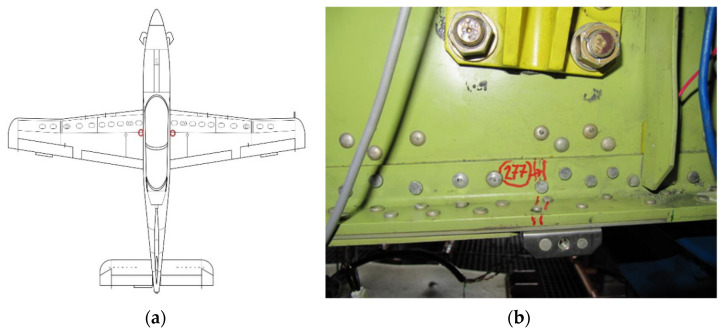
Location of the CP2 critical point on the wing main spar. (**a**) General location in the structure (LHS and RHS); (**b**) actual damage found during the test (damage No. 277 RHS) found after 21,000 SFHs.

The critical location of the PZL-130 “Orlik” trainer aircraft (CP2) was determined to be in the lower flange of the wing rear spar in the vicinity of rib No. 2 (Figure 3). It was one of the fatigue damages found during repetitive Non-Destructive Inspections (NDI) of the test specimen throughout the test. Such locations are addressed as the Critical Points (CP) and are usually defined during a FSFT. In the case of discussed damage, it was one of the defined CP in the PZL-130 “Orlik” TC-II structure as a final result of the FSFT. The CP in this case was defined by gathering all the damage found during the FSFT and the ten most severe ones in the primary structure, like wing spar and longerons, fuselage longerons etc., were selected as CP.

The damage considered within this article was the sole reason for the test to terminate finally since it was located in the primary structure on the lower flange of the rear spar. The total ten CP were defined at the end of the test, selected from the most significant damage found in significant structural elements of the aircraft structure. Some CP among the ten were selected in the vicinity of other CP since they were caused by the same load however were located in much more accessible regions, which made them more beneficial from the inspection point of view. The herein considered crack was a result of the bending moment acting on the wing during overload cycles (the main fatigue driver for light aircraft structures), causing tension in the lower flange. The tension combined with stress concentrations due to fasteners which connect the lower flange with the lower skin resulted in crack nucleation.

Moreover, the discrete load application, although a complex whiffle tree was applied, could have influenced the onset and propagation of the considered damage. The crack was found both on the left (LHS) and right-hand side (RHS) after 29,050 Simulated Flight Hours (SFHs) and 21,000 SFHs, respectively [24]. The SFHs are a representation of the Actual Flight Hours (AFHs) which are generally the real time operation loads of an aircraft. The main advantage of the FSFT is that it consists of all the fatigue significant loads but executed at a much faster pace than in the actual operation. This is achieved by filtering the actual loads representing AFHs (which gives the SFHs) and neglecting the fatigue insignificant loads, which take up a significant amount of time in a single operating hour.

Since the NDI findings during the FSFT provided little information about the actual propagation of the cracks, it was crucial to determine the crack propagation curves in order to establish possible inspection regimes so that the cracks could be found in the early stages of development during scheduled NDI.

### 2.2. Stress Intensity Factor Definition

Crack propagation calculations are based on the stress range induced by the loads exerted on the structure and thus the resultant stress intensity factor (*K*). This parameter is dependent on the geometry and loads applied to the structure. In this work the authors focus on the dimensionless stress intensity factor (*β*), which is a purely geometric feature of the structure and therefore does not depend on the actual loads applied. Dimensionless stress intensity factors for trivial and more complex—yet common—geometries can be found in the literature [25]. However, for more complex geometries the stress intensity factor must be determined individually. For this reason, a numerical representation of the structure with propagating crack is defined and the stress intensity values are defined along the crack’s length.

**Figure 4:**
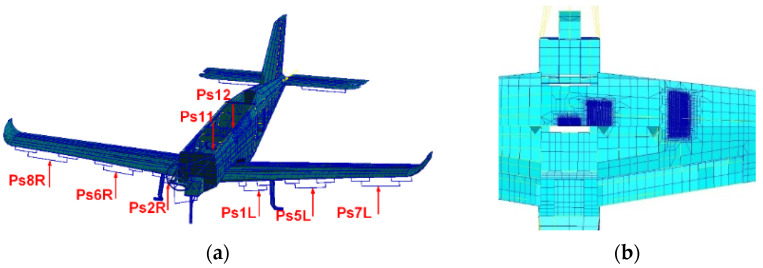
Global FEM model of the aircraft structure. (**a**) Load introduction according to the actual FSFT actuators layout; (**b**) local mesh densification (middle mesh) in order to obtain detailed strain fields for submodels (bottom view).

In order to determine β for the herein described analysis, the global FEM model (provided by the manufacturer of PZL-130 “Orlik”) was adjusted. Additional mesh densification was applied to the CP region (Figure 4b) in order to obtain detailed displacement fields to be used in the local models. The global model was defined in the MSC Software and composed of combined linear shell, beam and solid elements (Figure 4a). In order to obtain realistic load distribution, the model was loaded by means of concentrated forces using additional whiffle trees identically as in the FSFT (Figure 4a). To determine the stress distribution the horizontal flight (vertical overload equal to 1) load condition was used to define the stress field. Table 1 sets out the forces used in this load state. The displacement field was defined for the area corresponding to the local FEM model of the structure adjacent to the crack onset location (Figure 4b). The global-local FEM model approach was used [26,27,28,29] to apply denser mesh in the vicinity of the crack as well as crack propagation by separating consecutive nodes in the local model, whereas the global model was used only once for the local model boundary strain field definition. This opened the way for the most laborious calculations to be performed using only the local model.

**Figure 5:**
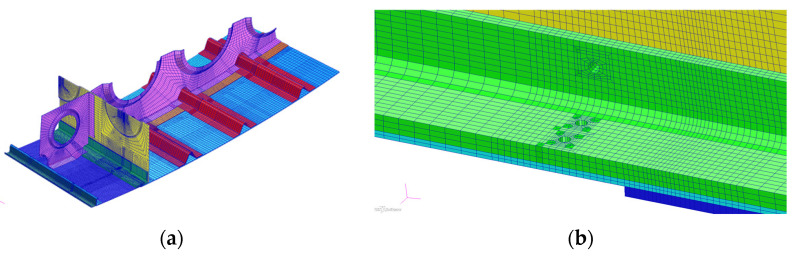
Finite element models of the damaged lower flange structure. (**a**) Rib No. 2 area; (**b**) rib No. 5 area.

The local FEM model of the CP2 consisted of 52,979 solid elements. The use of local submodel allows to reduce global computation time allowing for dense mesh in the region of crack propagation at the same time [26,27,28,29]. Figure 5a shows the general view of the whole local model, whereas Figure 5b shows the crack location in more detail. As mentioned earlier, the FEM models were developed based on detailed geometry delivered by the aircraft manufacturer using MSC Software products (MSC.Patran and MSC.Marc). Much care was taken on order to define mesh with proper element size, especially in the damaged location.

Material properties were identical with those used in further crack propagation calculations (Table 2) and were obtained throughout additional material test carried out on samples delivered by the manufacturer of the aircraft [21,30].

**Figure 6:**
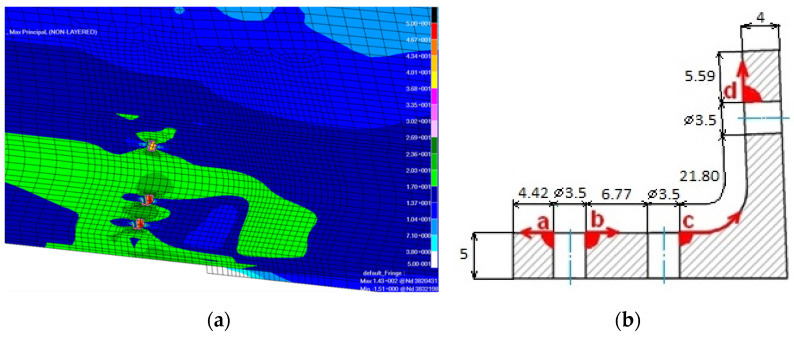
Crack initiation schedule for the region. (**a**) Stress concentrations from the FEM model; (**b**) assumed crack.

Figure 6a shows the maximum principal stresses in the vicinity of the fastener holes, which were crack initiators in the damaged element. Based both on the numerically obtained stress values, and visual inspection of the damaged element four crack sections were defined throughout the element:

- section a—from the first hole in the horizontal part toward the flange end,
- section b—from the first hole toward the second hole in the horizontal part,
- section c—from the second hole toward the hole in the vertical part,
- section d—from the hole in the vertical part toward the upper end.

Figure 6b shows the initial crack onsets (corner cracks) and the arrows depict assumed direction of crack propagation. The initial crack lengths are given as *a_i_* in Table 2.

Table 2 includes initial crack parameters for all considered sections as well as material properties of the 2024-T4 aluminum alloy, e.g., Young’s modulus (E), yield stress (σy) and ultimate stress (UTS) obtained from laboratory tests [21,30].

For the *β* definition, the Virtual Crack Closure Technique (VCCT) was used, according to which the energy released from cracking material (*G*) during an infinitesimally small increment is equal to the work needed to rejoin the crack on that increment, counteracting reaction forces in the nodes just in front of the crack [29,30,31]. Therefore, in order to establish the energy release rate, it is necessary to determine reaction forces in the nodes in the tip of the crack as well as the displacement of nodes preceding the crack tip. An additional assumption used in VCCT is that the reaction force in the neighboring nodes lying on the crack path is equal due to the small element size; therefore, *G* estimation can be made in one numerical step for a defined crack length.

**Figure 7:**
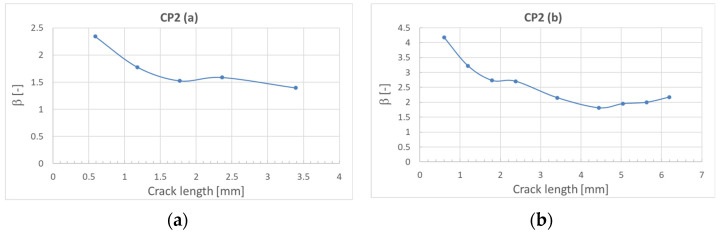
Calculated values of *β* versus crack length. (**a**) For section *a* of CP2; (**b**) for section *b* of CP2.

**Figure 8:**
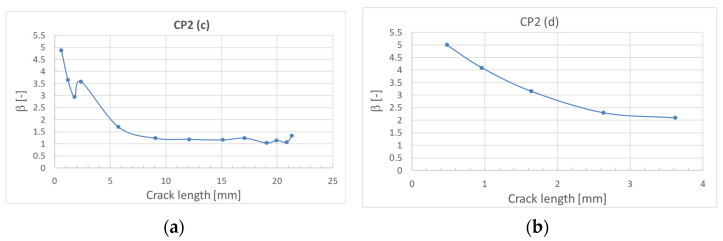
Calculated values of *β* versus crack length. (**a**) For section *c* of CP2; (**b**) for section *d* of CP2.

The VCCT module in MSC Patran (using the MSC. Marc solver) allows for *G* definition in a crack tip created by discontinuity in the mesh. In order to obtain the *β* it is necessary to use the following formula for stress intensity factor *K*, derived from the energy release rate and stress intensity factor relation [19,32]:

(1) $$K=\sqrt{\frac{GE}{\alpha }}$$

where *E* is the Young’s modulus of the material, whereas for the plane stress state:

(2) α=1,

and for the plane strain state:

(3) $$\alpha =1-\upsilon^{2},$$

where *υ* is the Poisson’s ratio of the material. Finally, *β* can be derived from calculated *G* values using the formula:

(4) $$\beta =\frac{K}{\sigma \sqrt{\pi a}}=\frac{1}{\sigma }\sqrt{\frac{GE}{\pi a\alpha }},$$

where *σ* is the bypass stress in the considered element section and *a* is the crack length. The values of *β* versus crack length were obtained by numerical calculations defining modeling the discontinuity in the element and defining the corresponding values of *G* and then calculating the *β* with the formula above. The results obtained for sections *a* and *b* are shown in Figure 7 and for sections *c* and *d* in Figure 8.

## 3. Load Spectrum Definition

Load spectrum used in the presented crack propagation estimations was based on the strain data recorded during the actual FSFT. The test was composed of two types of load spectra each representing 200 SFH [17]. The first type was repeated three times and then followed by one repetition of the second type. Therefore, the total load spectrum consisted of 800 SFH which were repeated throughout the test. In order to represent the actual load condition in the damaged spar it was essential to define load sequences for both spectrum types and then compose the 800 SFH load spectrum. As mentioned in the previous section, the rear spar strain gage was damaged during the test and it was not possible to gather full load information throughout both types of load spectra used in the test, therefore the actual load sequence was estimated basing on the front spar strain data correlated with the available rear spar strain data. Both strain gages recorded tension in lower wing spar flanges due to wing bending and therefore correlated very well.

**Figure 9:**
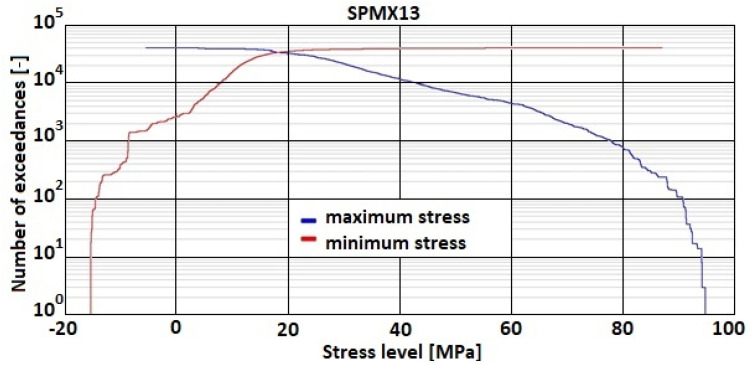
Exceedance plot of the defined load spectrum based on the strain gage data.

The recorded strain signal was defined in μStr; however, basing on the laboratory material test data [30] and assuming elastic material behavior in the strain gage installation region the strain values were transformed to stress units and presented in a form recognized as load spectrum in the AFGROW software [22]. The overall spectrum is shown in Figure 9 in the form of an exceedance plot showing number of cycles exceeding minimal and maximal stress values shown correspondingly in blue and red.

## 4. Crack Propagation Curves Definition

The *β* values obtained from the numerical calculations had to be tabularized for use in AFGROW software [22]. Since the load spectrum used both in the FSFT and in the presented analysis was a flight-by-flight variable amplitude load spectrum, the retardation effect had to be considered.

**Figure 10:**
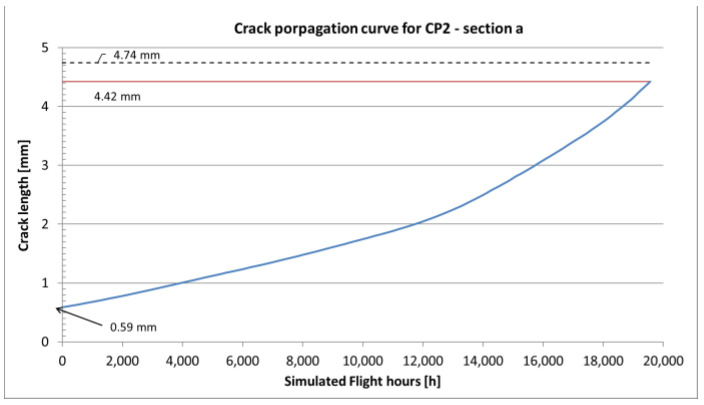
Crack propagation curve and inspection intervals definition for section *a* of CP2.

**Figure 11:**
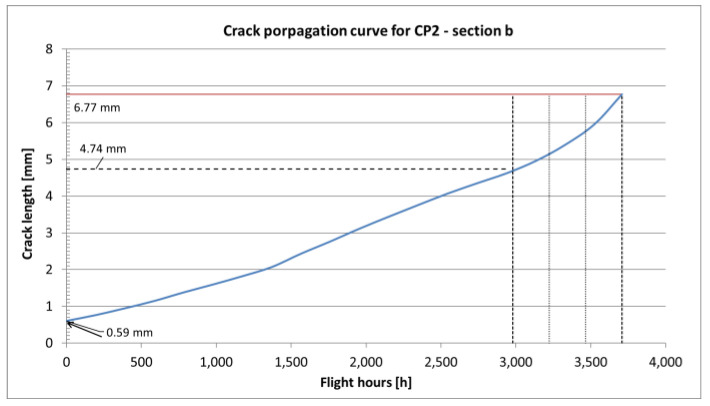
Crack propagation curve and inspection intervals definition for section *b* of CP2.

**Figure 12:**
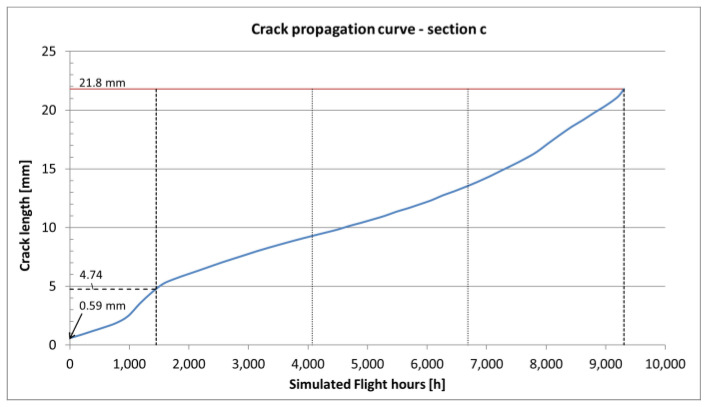
Crack propagation curve and inspection intervals definition for section *c* of CP2.

**Figure 13:**
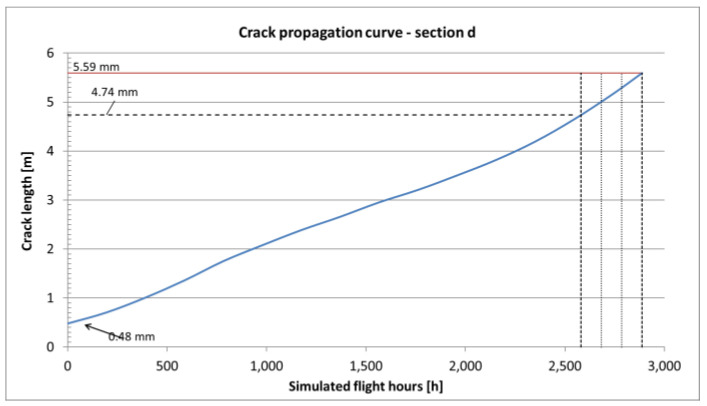
Crack propagation curve and inspection intervals definition for section *d* of CP2.

In the case of variable amplitude load spectra, especially when high amplitude loads are present, the retardation effect can be observed. Higher loads cause creation of larger local residual stresses in the crack tip plastic zone region, which have to be overcome by the following cycles in order for the crack to continue propagating. The retardation effect was taken into account using the Willenborg model [19] and for aluminum alloys the retardation is assumed to be 3 [22], which relates the residual plastic stress with the stress induced with the overload cycle. The final obtained crack propagation curves for all the *a*, *b*, *c* and *d* sections are shown in Figure 10, Figure 11, Figure 12 and Figure 13.

## 5. Discussion

The carried-out analysis process and the final crack propagation calculations allowed for definition of the crack propagation curves along four consecutive sections of the lower flange damaged during the FSFT.

The results of crack propagation estimations are shown in Figure 11, Figure 12 and Figure 13. Each curve starts with the assumed initial crack size and the crack length versus Simulated Flight Hours (SFH) is given on the graphs. In each figure, the dashed line corresponds with the detectable crack size (about 4.74 mm for a surface crack in a flat element inspected with Eddy Currents). The red line corresponds with the critical crack size, which is the crack size for which the element is no longer capable to carry the exerted load (this condition is checked by the computing program with each pass of the load spectrum for the given material data) and the element fails. Intersection of the estimated crack curve with both aforementioned lines is the actual crack propagation region, which is interesting in terms of maintenance.

Within this regime, NDI can result in crack-finding and estimation of the actual length of a crack. The other important factor of an NDI is the Probability of Detection (POD), which is a complex phenomenon resulting from many aspects like the human factor, accessibility of the inspection site, type of damage and the method used during inspection. To mitigate the influence of mentioned limitations, it is advised to define NDI in such a way that the crack can be found at least twice during the time between detectable and critical crack size [33]. Following this approach, the time interval between the time when the crack reaches detectable crack length and time when it reaches critical size is divided in three. The obtained time interval is the time between the initial inspection (around the time a crack reaches detectable crack size) and the following recurring inspections.

The total time of each crack propagating separately one after another in the assumed order is 35,515 SFHs. The severed lower flanges were found after 21,000 SFHs for the right-hand side and after 29,050 SFHs for the left-hand side. The total life obtained from the presented analysis slightly exceeds the time of findings. However, it is possible that the cracks in the following sections initiated before cracks reached critical values in the previous sections, as assumed in the presented analysis. The estimated recurring inspection intervals for the four considered sections are:

- for section a—the critical crack size is smaller than the detectable crack size,
- for section b—the inspection interval would be 231 h,
- for section c—the inspection interval would be 2622 h,
- for section d—the inspection interval would be 101 h.

In general, the obtained crack propagation curves show a stable rate of propagation, however the expected time intervals between detectable and critical crack lengths for sections *b* and *d* as well as critical crack size smaller, then detectable crack size for section *a* make them unsuitable for damage tolerance. Only section *c*, with its stable crack propagation and relatively long intervals, is suitable for damage tolerance approach.

The relatively short life of section *a* makes it unsuitable for damage tolerance, but the residual strength of the adjacent structure makes section *a* a good indicator of the onset of fatigue damage in this region. As expected, due to the overall thickness and geometry of the element, the most durable section was section *c*, with a total fatigue life of 9305 SFHs and an inspection interval of 2622 h, which is a reasonable interval from the operation point of view.

The assumed nondestructive inspection technique is Eddy Current inspection using a portable detectoscope with a handheld probe. Such equipment is usually available at the depot and could be used to verify the presence of cracks during regular inspections. In order to verify the findings and to define precisely the actual crack length more sophisticated inspection procedures can be used [34], but this would increase the inspection burden and should be performed during more complex services.

From the maintenance point of view, the most convenient solution would be to perform regular simplified EC inspections at depots and more complex tests including paint stripping during overhauls. The results obtained will provide insight for inspection of the CP2 during operation of the PZL-130 “Orlik” fleet, but the methodology may be subject to change on the basis of actual operation findings.

## 6. Conclusions

Presented results were obtained using strain data recorded with an onsite strain gage during FSFT of the PZL-130 “Orlik” military trainer aircraft. Using direct strain measurements during FSFT to define the load spectrum for calculations allowed to take into account all the possible local effects coming from discrete load application during FSFT as well as local stress conditions. The load spectrum, combined with dimensionless stress intensity factor *β* obtained from FEM local model using VCCT and energy release rate definition and material tests carried out on samples delivered by the aircraft manufacturer, allowed for crack propagation curves estimation for the damaged element.

The obtained curves were used to estimate both time of initial NDI (after the cracks will reach detectable crack sizes) as well as recurring NDI inspections (scheduled in such way, that the curve would be found at least once between detectable and critical crack size). Due to location of strain gauges during the FSFT, it was possible to evaluate the actual load spectrum for the cracked element (not estimating it from the general load condition of the wing), which makes the results more reliable and unique. However, it is essential to be aware of the possible local stress concentrations (especially in a riveted or geometrically complex structure). For the purpose of crack propagation, the general bypass stress, uninfluenced by local concentrations should be applied. This could be achieved by installing the strain gauge as far as possible from any stress concentrators or using smaller grid sizes.

The herein presented analysis was used as part of the final results of the full-scale fatigue test of the PZL-130 “Orlik” aircraft. Obtained results corresponded well with the NDI findings during the test, however crack propagation estimations are result of many input data and with a slight change in any of them different results may be obtained. Further research will be devoted in sensibility analysis of the crack propagation calculations in order to define how the input data affect the final results. Moreover, the influence of load spectrum, in particular data losses resulting from human error and hardware malfunction, will be investigated.

## Figures and Tables

**Table 1:** Values of forces used for strain field definition in the global FEM model.

Force	Ps1L	Ps2R	Ps5L	Ps6R	Ps7L	Ps8R	Ps11	Ps12
Value [N]	1883	1883	2648	2648	2730	2730	−1521	−1521

**Table 2:** Cracks initial parameters and material properties of the considered 2024-T4 aluminium alloy.

Parameter	CP2_a	CP2_b	CP2_c	CP2_d	Unit
Length	4.42	6.77	21.8	5.59	[mm]
Thickness	5	5	5	4	[mm]
a_i_	0.59	0.60	0.60	0.48	[mm]
K_IC_	36.75	36.75	36.75	36.75	[MPa√m]
E	72 000	72 000	72 000	72 000	[MPa]
σ_y_	319	319	319	319	[MPa]
UTS	469	469	469	469	[MPa]
SMF	0.072	0.072	0.072	0.072	[-]
